# Umbilical Sepsis

**Published:** 1897-06-05

**Authors:** 


### UMBILICAL SEPSIS.

That the treatment of the cord in the new-born child is a matter of very considerable importance every one will admit, although it is to be feared that it is too often dealt with as if it were but of small moment. Now and again, however, one hears of outbreaks of disease arising from infection of the funis, which show how desirable it is that the wounded cord should be treated as carefully as any other wounded structure, although not, perhaps, precisely in the same way.

The tale of the "scourge of St. Kilda," the prevalence of tetanus neonatorum, by which those islands have been saved from having to deal with the problem of over-population, is well known. Dr. Arthur Mitchell, writing in 1865, says that out of 125 children, the offspring of the 14 married couples residing on the island in 1860, no less than 84 died within the first 14 days of life, or, in other words, 67·2 per cent. Perhaps it is not so well known how completely this terrible mortality has been checked by the simple process of ensuring that the new-born infants should be wrapped in clean flannel, and that the navel should from the first be dressed with iodoform. The islanders have to thank the Rev. Angus Fiddes, the minister of St. Kilda, for coming over to the mainland and consulting various authorities as to the best course to pursue to diminish this great mortality, with the result that he was able to report, in 1894, that he had not lost a single case since he had begun to use this treatment.

During the year 1896 there has been a considerable prevalence of umbilical sepsis in the Nursery and Child's Hospital in New York, five children dying of the disease out of the 169 births which occurred in the institution during the year, besides a large number who suffered more or less severely, fever having occurred in 95 of the cases.

Locally this disease may show itself by erysipelas, cellulitis, gangrene, lymphangitis, phlebitis, or arteritis, while generally the symptoms may take the form of fever, loss of weight, jaundice, skin eruptions, gastro-enteritis, abscess, and thrombosis of the veins or arteries. It would seem, however, from the published account that the local symptoms in the fatal cases were comparatively slight, and when one reads of pyrexia and loss of weight with diarrhœa being the main symptoms, while very little was to be seen wrong with the cord itself, one cannot but entertain the suspicion that in not a few of the instances which we hear of in which death is returned as occurring within the first ten days of life from various conditions of innutrition, the real cause may have been umbilical sepsis.

In the treatment of the funis it is well to remember that one of the most important natural means for preventing the occurrence of sepsis is the drying and shrivelling up which the cord quickly undergoes. To wrap it up, then, in many layers of any material, and especially to dress it with powders which, after coming in contact with moisture, dry into hard, impervious coatings, through which evaporation cannot well take place, is a mistake. The stump of the cord may be dabbed with solution of perchloride of mercury, and wrapped up in a thin layer of sterilised absorbent cotton wool or gauze, or, perhaps even better still, in the traditional singed linen; it may then be turned up on the abdomen, so as to keep it away from the influence of the urine, and kept in place by a turn of flannel bandage, but no such excess of any form of wrapping should be employed as to interfere with evaporation and the desiccating process, which is so essential to the proper separation of the cord, and no powder should be used. It is not uninteresting to note that the traditional piece of singed linen, fresh from the fire, which dates from so far back, is really a sterilised dressing, and that the "old woman's tale," that the child will die if the cord is turned downwards, is but an expression of the fact that if the stump gets wet septic mischief is more likely to occur.

